# Longevity and growth of *Acacia tortilis*; insights from ^14^C content and anatomy of wood

**DOI:** 10.1186/1472-6785-7-4

**Published:** 2007-06-15

**Authors:** Gidske L Andersen, Knut Krzywinski

**Affiliations:** 1 Department of Biology, University of Bergen, P.O.Box 7800, N-5020 Bergen, Norway

## Abstract

**Background:**

*Acacia tortilis *is a keystone species across arid ecosystems in Africa and the Middle East. Yet, its life-history, longevity and growth are poorly known, and consequently ongoing changes in tree populations cannot be managed in an appropriate manner. In other arid areas parenchymatic bands marking growth zones in the wood have made dendrochronological studies possible. The possibilities for using pre- and post-bomb ^14^C content in wood samples along with the presence of narrow marginal parenchymatic bands in the wood is therefore tested to gain further insight into the age, growth and growth conditions of *A. tortilis *in the hyper-arid Eastern Desert of Egypt.

**Results:**

Based on age scenarios and the Gompertz growth equation, the age of trees studied seems to be from 200 up to 650 years. Annual radial growth estimated from calibrated dates based on the post-bomb ^14^C content of samples is up to 2.4 mm, but varies both spatially and temporally. Parenchymatic bands are not formed regularly. The correlation in band pattern among trees is poor, both among and within sites.

**Conclusion:**

The post-bomb ^14^C content of *A. tortilis *wood gives valuable information on tree growth and is required to assess the age scenario approach applied here. This approach indicates high longevities and slow growth of trees. Special management measures should therefore be taken at sites where the trend in tree population size is negative. The possibilities for dendrochronological studies based on *A. tortilis *from the Eastern Desert are poor. However, marginal parenchymatic bands can give insight into fine scale variation in growth conditions and the past management of trees.

## Background

*Acacia tortilis *is a keystone species growing across arid ecosystems in Africa and the Middle East, from moist savannas to hyper-arid deserts. It is of importance for people and their domesticated animals, improves soil fertility and increases biodiversity [1-4]. It is well adapted to disturbances such as drought, fire, browsing and pollarding. In spite of its ecological importance and wide distribution, knowledge about its long-term dynamics, including essential life historical characteristics such as longevity and growth is poor. This is also true of other *Acacia *species in this region and of arid land trees in general [5-9]. From a population dynamics perspective and seen in relation to ongoing vegetation changes in arid lands, this knowledge is essential to develop sustainable management strategies.

In the area considered here, the hyper-arid Eastern Desert of Egypt (ED), no studies have attempted to estimate the longevity and growth of *A. tortilis*. Local nomads say that trees grow slowly and date to "Roman" times, i.e. the pre-islamic period [1]. This view is rendered credible by characteristics such as its great ability to resprout and its investment in defensive characters such as spines and dense, hard wood impregnated with resins and crystals. Slow growth is indicated by the results of a change analysis based on historical imagery (1965) and field observations (2003) [10]. Seen from a life historical perspective such characteristics are often associated with great longevity [11,12].

If trees are old, one might wonder if they are relicts from periods when moisture conditions were more favourable for recruitment. Determining the age of tropical and subtropical trees is, however, problematic. In temperate climates annual growth rings are formed in response to climatic seasonality. Anatomically, these rings are easily detectable because of distinct seasonal differences between early- and late-wood. In the tropics and sub-tropics, seasonality is less distinct and growth rings, if present, are of a different anatomical type which can be more difficult to detect [13]. Nevertheless, annual growth rings are known from a substantial number of tropical species [14].

In *A. tortilis *a narrow marginal parenchymatic band associated with crystalliferous calcium oxalate chains is present in the wood [7,15], and according to these studies they are formed coincidentally with a temporary pause in growth. Such bands are also found in *A. tortilis *wood from the ED. If these bands are formed regularly, they are internal time markers that can facilitate age determination [5]. If regularity is annual, standard dendrochronological methods might be applied to *A. tortilis *[7,16]. However, even if bands don't provide any chronological information, correlations between individual band patterns are of interest because they mark times when conditions did not permit growth. As such, patterns of bands may provide insight into the spatial variability of growth conditions and consequently into the factors affecting the growth of trees.

Another method providing insight into tree age and growth is radiocarbon dating. ^14^C content is a temporal marker in wood and has been successfully used for dating of wood and to confirm ages or time intervals estimated from ring counts. For wood older than AD 1950, conventional radiocarbon ages estimated as years Before Present (0 BP = AD 1950) are calculated on the basis of the Libby half-life time (5568 years) [17]. The radiocarbon age is calibrated to calendar date (BC/AD) based on estimates from dendrochronologically dated tree ring samples [18]. Because the level of ^14^C in the atmosphere has not been constant over time, there are variations in the calibration curve, including a number of plateaus. The wiggly plateau period between 1650 and 1950 makes tree age estimation difficult since the ^14^C content of a sample from this period suggests more than one calendar date.

After 1950 the level of^14^C in the atmosphere rose to very high levels as a result of the Cold War nuclear bomb testing. After a few years of exceptionally rapid increase from the late 50s, this post-bomb ^14^C signature curve reached a peak in 1964, thereafter the ban on nuclear bomb testing has caused a slow decrease up to present [19,20]. This post-bomb ^14^C makes accurate dating possible and has been successfully used to determine the annual character of rings in tropical trees [14,21].

In the present study we test the possibility of using pre- and post-bomb ^14^C in wood samples along with the presence of narrow marginal parenchymatic bands in the wood to gain further insight into the age, growth and growth conditions of *A. tortilis *in the hyper-arid ED.

### Study area

**Figure 1:**
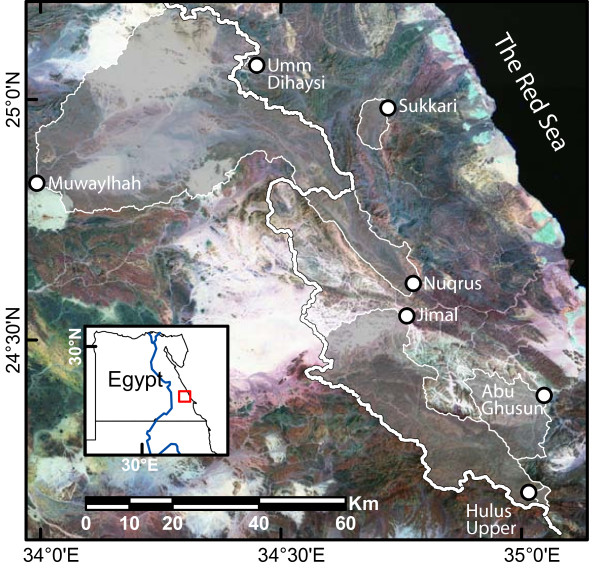
**Study area**. Sites from which cores were sampled, seen in relation to their catchments and east-west water divide. A Landsat TM image is displayed in the background.

**Figure 2:**
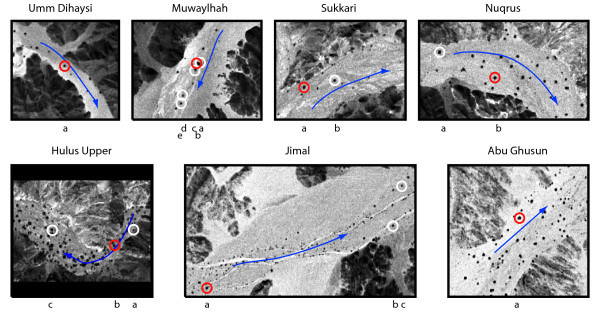
**Sites studied and trees sampled**. Sampled individuals are encircled on imagery from the CORONA satellite (KH-4B, 1971), where trees are seen as black dots. Red symbols encircle individuals from which increment cores were dated. White symbols encircle other sampled individuals. Arrows indicate direction of water run-off. Letters refer to core name; named from up- to down- stream.

Material has been collected in the ED (Figs. 1 and 2) from individuals of *Acacia tortilis *(Forssk.) Hayne [22]. Two subspecies occur in the ED and those sampled here are ssp. *raddiana *[22]. However, we refer to *A. tortilis *at the species level because gradual morphological transitions are found and desert dwellers shape the very morphology of trees (see below).

*A. tortilis *is the dominant tree species in the mountainous landscape of the ED and is confined to seasonally dry river-valleys, *wadis*, where soil moisture conditions permits its establishment and continued growth. Rainfall (< 30 mm/year; [23]) is temporally variable, rare and spatially scattered. Moisture conditions vary among individual catchments and drainage systems. Surface water and soil moisture in the upper part of the soil is highly unreliable and trees and other perennial vegetation seem therefore to rely mainly upon deeper and permanent soil moisture during the long dry periods between rains [24,25].

Except for water, important factors influencing tree growth are branch cutting, i.e. pollarding, and extensive browsing by wild and domesticated animals. Both are widespread traditional management strategies controlled by the pastoralists, and are securely documented in the ED at least back to ca. 3000 BP [2]. Pollarding is performed on all mature trees and contributes not only to the characteristic growth form of *A. tortilis*, but may even stop growth temporarily. Browsing significantly retards growth, particularly in the early stages of a trees' life [26].

## Results

### ^14^C analyses

**Figure 3:**
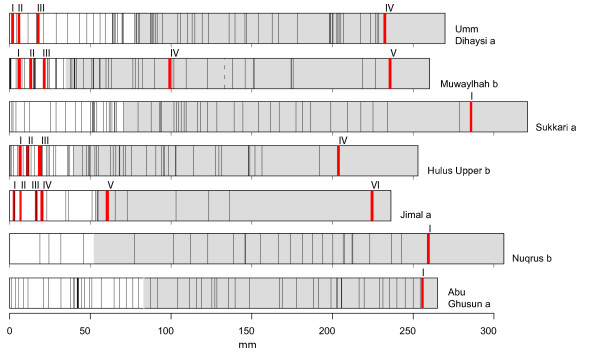
**Sketch of dated cores**. Red areas indicate sample location. Black lines are parenchymatic bands (appear as black areas when several rings are close together). The white part of the core is sapwood, the grey is heartwood. Dashed lines indicate a gap in the core.

**Figure 4:**
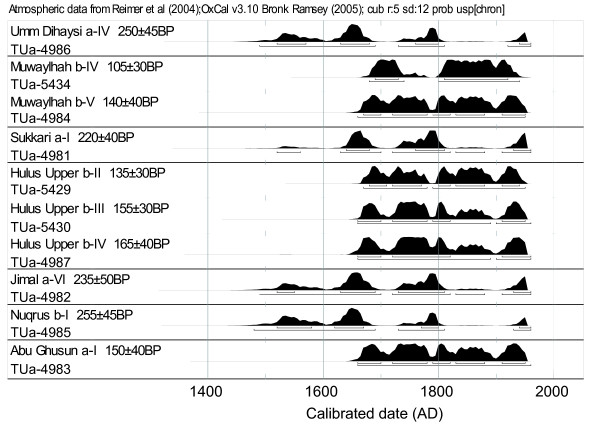
**Probability distributions of calibrated dates for samples older than AD 1950**. The radiocarbon age gives ambiguous calibrated dates for all samples; i.e. wood is formed in the period between 1650 and 1950. Samples with conventional radiocarbon ages of more than 200 years might have been formed as far back as the 16^th ^century. Roman numerals refer to locations of samples in the core (Fig. 3). Horizontal lines below probability distributions represent the 1 (upper) and 2 sigma ranges of calibrated dates.

All the oldest wood samples (Fig. 3) have a ^14^C content too high to give a unique calibrated date (cf. Background, Fig 4). The wood in most samples was formed between 1650 and 1950. However, for samples with ^14^C ages greater than 200 years, it is possible that the wood was formed as far back as the 16^th ^century.

Calibrated dates and subsequent growth rates for hyperactive (i.e. post-bomb levels) samples are reported in Table 1. For a few samples calibrated dates are incompatible or ambiguous. For *Umm Dihaysi a*, sample I may be contaminated by older material and is therefore disregarded here. Calibrations of samples II and III give a set of two similar dates. The most likely dates are 1998 (II) and 1956 (III), when smoothing, combination of suggested dates and subsequent growth rates are taken into consideration. For *Jimal a*, samples III and IV have about the same possible range (ca. 1991–1993). Since sample IV is older than III, we use 1991 (IV) and 1993 (III) to estimate growth rates. For sample V two dates are possible (pre- or post- 1964), both giving reasonable growth rates, but 1963 is less likely than 1970.

**Table 1:** Dating results for hyperactive samples.

Core	Sample	Lab. Ref.	F14C	+/-	δ13C	Calibrated date	Intervening GR	Overall GR
Umm Dihaysi a	I^1^	TUa-5425	1.045	0.004	-25.2	-	-	
Umm Dihaysi a	II	TUa-5426	1.080	0.005	-25.6	1998.1 (1956.7)	1.13 (0.13)	
Umm Dihaysi a	III	TUa-5427	1.090	0.005	-24.2	1956.5 (1996.2/1997.4)	0.29 (6.18/15.60)	0.38
Muwaylhah b	I	TUa-5431	1.130	0.004	-28.8	1993.9	0.63	
Muwaylhah b	II	TUa-5432	1.158	0.005	-28.4	1990.2	1.93	
Muwaylhah b	III	TUa-5433	1.303	0.005	-27.0	1979.3	0.77	0.89
Hulus Upper	I	TUa-5428	1.381	0.006	-24.3	1975.5	0.23	0.23
Jimal a	I	TUa-5420	1.082	0.005	-27.4	1998.2	0.52	
Jimal a	II	TUa-5421	1.107	0.005	-25.5	1996.8	0.66	
Jimal a	III^1^	TUa-5422	1.143	0.005	-26.7	1992.8	2.37	
Jimal a	IV^1^	TUa-5423	1.141	0.005	-27.4	1991.3	2.39	
Jimal a	V	TUa-5424	1.514	0.007	-25.7	1971.0 (1963.0)	2.00 (1.44)	1.87

^14^C content (F14C, including measurement error (+/-) and fractionation values (δ13C)), calibrated dates (1 sigma midpoint) and estimated growth rates (GR in mm/yr) are summarised. The intervening GR is estimated, based on the period between the sample under consideration and the next youngest sample. Overall GR is estimated based on the period between the oldest date and sample date (2003).

^1 ^For ambiguous dates refer to the text for further explanation.

The calibrated dates of hyperactive samples show that growth after 1950 varies both temporally and spatially (Table 1). Based on the oldest hyperactive calibrated dates, the overall growth is 8 times faster at Jimal than at Hulus Upper. Great variation in growth is evident within all cores where more than one sample is dated.

There is no indication in the material that bands can be taken as regular time markers within the core, and bands cannot therefore be used for dating trees (Adjusted R^2^: 0.04, F-statistic: 1.43 on 1 and 9 DF, p-value: 0.26). For *Jimal a *there is an apparently significant relationship between the number of bands and the time period in which they were generated (Adjusted R^2^: 0.99, F-statistic: 449.4 on 1 and 3 DF, p-value < 0.001), suggesting that one band was formed every 2.2 years. However, data are far too few to generalize. This relationship is rejected since it would suggest an age of 35 years (15 bands) and consequently that the inner sample (VI) is hyperactive, which is not the case (Fig. 3).

### Age scenarios

**Figure 5:**
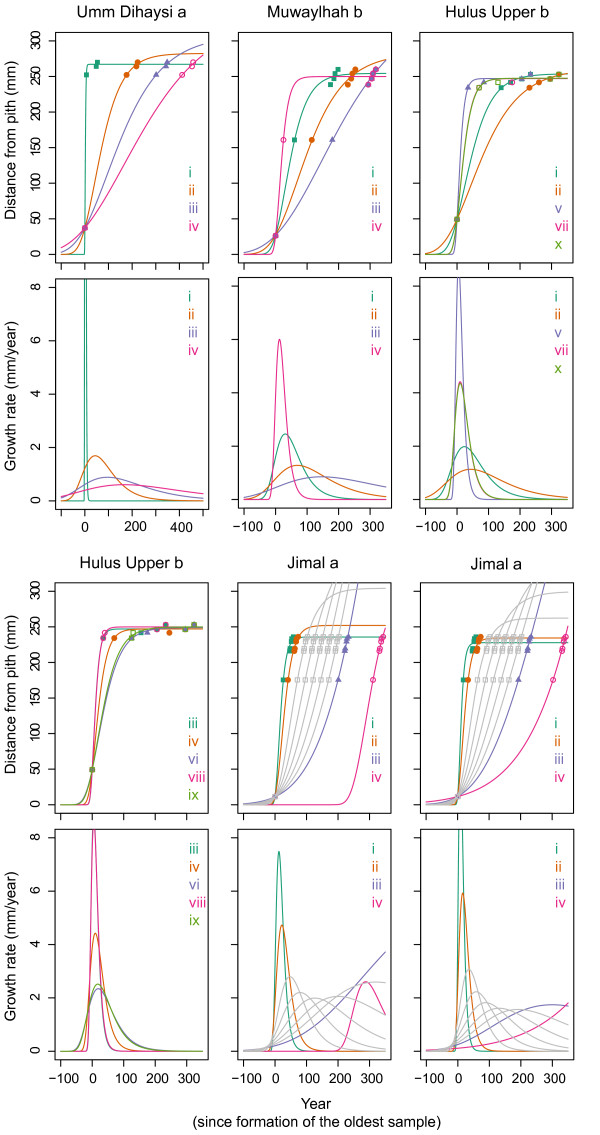
**Plots of age scenarios**. The upper panel shows cumulative growth, and the lower panel differential growth as modelled by the Gompertz equation. The oldest sample is by definition formed in year 0. Establishment is defined as the year in which cumulative radial growth is at least 1 mm, and will be negative compared to the oldest sample from the core in question. Scenario names refer to Table 2. Grey lines (for Jimal a) show scenarios based on 25-year interval dates within 2 sigma ranges for sample VI, i.e. 1800, 1825, 1850, 1875 and 1900. Note the different x-scale on Umm Dihaysi a.

Age scenarios (see Methods) used to approximate the age of trees are presented in Table 2 and Fig. 5.

**Table 2:** Summary of age scenarios.

Core	Scenario	Date combinations						Gompertz parameters			Establ.
		I	II	III	IV	V	VI	a	b	c	

Umm Duhaysy a	i	-	1998.1	1956.5	1950			267.1	1.971	*0.545*	1948
Umm Duhaysy a	ii	-	1998.1	1956.5	1780			282.4	2.027	*0.016*	1719
Umm Duhaysy a	iii	-	1998.1	1956.5	1655			308.0	2.113	*0.008*	1528
Umm Duhaysy a	iv	-	1998.1	1956.5	1545			*342.0*	2.218	*0.005*	1346
Muwaylhah b	i	1993.9	1990.2	1979.3	1865	1805		254.4	2.244	0.026	1772
Muwaylhah b	ii	1993.9	1990.2	1979.3	1865	1750		282.6	2.346	0.012	1680
Muwaylhah b	iii	1993.9	1990.2	1979.3	1865	1685		355.2	2.585	0.007	1561
Muwaylhah b	iv	1993.9	1990.2	1979.3	1710	1685		249.9	2.254	0.065	1673
Hulus Upper b	i*	1975.5	1940	1910	1770			253.8	1.638	0.021	1714
Hulus Upper b	ii*	1975.5	1940	1910	1680			261.1	1.667	0.012	1579
Hulus Upper b	iii	1975.5	1925	1805	1770			247.1	1.612	0.097	1759
Hulus Upper b	iv	1975.5	1925	1750	1680			247.1	1.612	0.049	1656
Hulus Upper b	v	1975.5	1855	1805	1770			247.2	1.612	0.097	1759
Hulus Upper b	vi	1975.5	1855	1805	1680			249.1	1.618	0.026	1634
Hulus Upper b	vii	1975.5	1855	1750	1680			247.1	1.612	0.049	1656
Hulus Upper b	viii	1975.5	1810	1805	1770			249.9	1.623	0.094	1758
Hulus Upper b	ix	1975.5	1810	1805	1680			250.0	1.624	0.027	1637
Hulus Upper b	x	1975.5	1810	1750	1680			247.5	1.613	0.048	1636
Jimal a	i	1998.2	1996.8	1992.8	1991.3	1971.0	1945	235.7	2.915	0.086	1939
Jimal a	ii**	1998.2	1996.8	1992.8	1991.3	1971.0	1930	252.1	3.024	0.051	1920
Jimal a	iii	1998.2	1996.8	1992.8	1991.3	1971.0	1770	*4761.0*	6.052	0.003	1661
Jimal a	iv	1998.2	1996.8	1992.8	1991.3	1971.0	1660	*319.4*	*595.300*	*0.022*	-
Jimal a	v	1998.2	1996.8	1992.8	1991.3	1971.0	1535	-	-	-	-
Jimal a	vi^a^	1998.2	1996.8	1992.8	1991.3	1963.0	1945	227.8	2.937	0.134	1942
Jimal a	vii**^a^	1998.2	1996.8	1992.8	1991.3	1963.0	1930	234.4	2.923	0.069	1922
Jimal a	viii^a^	1998.2	1996.8	1992.8	1991.3	1963.0	1770	954.8	4.417	0.005	1683
Jimal a	ix^a^	1998.2	1996.8	1992.8	1991.3	1963.0	1660	*719500.0*	*11.050*	*0.001*	1448
Jimal a	x^a^	1998.2	1996.8	1992.8	1991.3	1963.0	1535	-	-	-	-

Date combinations are based on calibrated calendar dates (midpoints of 1 sigma ranges) of ^14^C dated samples. Some scenarios use other estimates: '*' lower (sample III) and upper (sample II) 1 sigma range, '**' lower 1 sigma range (sample VI). ^'a' ^indicates that the alternative date for sample V is used (see Table 1). Gompertz parameters in italics are not significant (p > 0.05). '-' indicates parameters that couldn't be estimated. Establishment date (Establ.) is defined as the year in which cumulative radial growth was at least 1 mm (Fig. 5).

For *Umm Dihaysi a *three scenarios are plausible, suggesting an establishment date around either 1700 (ii), 1550 (iii) or 1350 (iv). Except for the latter, the same approximate dates are likely for *Muwaylhah b *(scenarios ii and iii), but the fit is best for scenario iii. For *Hulus Upper b *most of the scenarios propose too fast growth over a continuous period compared to overall recent growth. The two remaining scenarios (i and ii) again suggest establishment either around 1700 or 1550; the oldest scenario is, however, in better agreement with recent growth, and the asymptotic value shows greater potential for continued growth. For *Jimal a *four of the scenarios seem impossible: scenario iii, viii and ix because of high asymptotic value (the largest tree observed in this part of the ED has a radius of 444 mm), and scenario iv because of the misfit between the calibrated age of the oldest sample in the scenario and its predicted age. The growth equation could not be fitted to scenario v and x. Of the remaining scenarios (i, ii, vi and vii; establishment early 1900), all predict a very abrupt cessation in growth. Consequently, we also considered 2-sigma ranges for sample VI which suggested a possibility for establishment between 1830 and 1880 (Fig. 4). Therefore we fitted additional scenarios, based on 25-year interval dates from 1800 to 1900 for the oldest sample (Fig. 5; *Jimal a*, grey curves), on this basis we suggest a likely establishment for *Jimal a *early 1800.

### Growth conditions and band patterns

**Figure 6:**
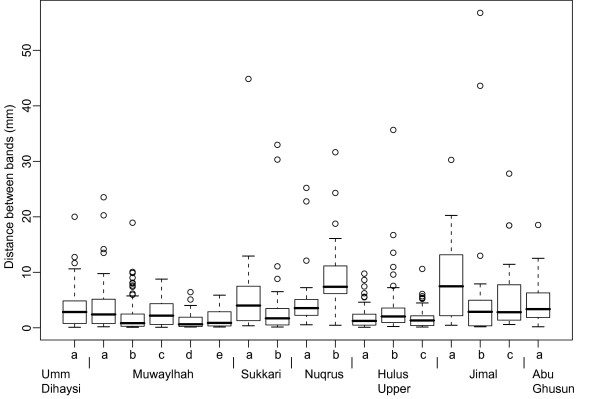
**Boxplot of distance between bands**. Distance between the narrow parenchymatic bands for all investigated cores.

Distances between bands vary among cores (Fig. 6). At Hulus Upper there is generally a short distance between bands (mean 1.7 – 3.6 mm). The same is seen in some of the Muwaylhah cores. A few cores have long distances between bands, e.g. *Sukkari a*, *Nuqrus b *and *Jimal a*. Several cores have a few bands that are far apart; often near the core pith. In some cores, however, bands might have been overlooked in certain parts because of disturbing knots (*Jimal c*, *Nuqrus b*, *Sukkari a *and *Muwaylhah a*) or because of slight and patchy wood decay (*Jimal a *and *b*; *Nuqrus a *and *Muwaylhah a *and *e*).

Correlations (expressed as t-values) between band patterns of cores within sites are not greater than those among sites (t = -1.02, df = 34.996, p-value = 0.84). Correlation coefficients are generally low, with the mean for both groups around 0. Within sites only two sets of cores were significantly positively correlated (p < 0.05; *Sukkari a *&*  b*; r = 0.32; *Jimal c & b*; r = 0.46), while among sites six sets of cores were significantly positively correlated (p < 0.05): *Umm Dihaysi a *&*Sukkari b *(r = 0.19), *Hulus Upper c *&*  Abu Ghusun a *(r = 0.23), *Nuqrus a *&*  Jimal a *(r = 0.52), *Sukkari b *&*  Nuqrus a *(r = 0.26), *Abu Ghusun a *&*  Jimal b *(r = 0.30) and *Muwaylhah b *&*Umm Dihaysi a *(r = 0.23).

The low correlations within sites suggest that factors operating at a local level influence growth conditions and band formation. We tried to remove these local effects within sites by correlating only shorter segments of cores, testing also for shifts of segments in either direction along the core to account for missing/false bands. As expected, within sites the proportion of significantly correlated segments at p < 0.05 decreases with longer segment lengths (mean 10 bands: 0.39; 15 bands: 0.32; 20 bands: 0.31) however, the difference is insignificant (Anova: Pr (>F) = 0.54). Also among sites the differences caused by segment lengths are insignificant (mean 10 bands: 0.36; 15 bands: 0.38; 20 bands: 0.39; Anova: Pr (>F) = 0.88). Further, the proportion of significantly correlated segments within sites at segment length 10 bands is not significantly higher than among sites (Anova: Pr (>F) = 0.59). This indicates that growth conditions are as variable within sites as among sites at time scales down to the time period represented by the shortest segment lengths (10 bands).

More than 50% of the segments tested (length 10) are significantly positively correlated for the following sets of cores (p < 0.05); within sites: *Hulus Upper c *&*  b*, *Jimal c *&*b*, *Sukkari b *&*a *and *Muwaylhah b *&*d *and among sites: *Umm Dihaysi a *&*Sukkari b*, *Umm Dihaysi a *&*Muwaylhah d*, *Hulus U b *&*Jimal b*, *Abu Ghusun a *&*Jimal b*, *Abu Ghusun a *&*Jimal c*.

## Discussion

Our results suggest that *A. tortilis *in the ED grows slowly and that some of the individuals studied could date back to AD 1550, one individual even as far back as AD 1350. The wiggly plateau in the calibration curve between 1650 and 1950 makes accurate estimates of tree longevity based on radiocarbon measurements difficult. We have attempted to obtain probable age estimates by fitting age scenarios that exploit all available and compatible growth rate and date information to the sigmoid Gompertz growth model. This model describes well the long-term trend in tree growth [27]. Growth rate is represented by a bell-shaped curve; i.e. growth rate increases at the beginning and decreases later in the lifetime of the tree. We applied this model because it represents the growth trend in *A. tortilis *better than a linear model of constant radial growth; a recent overall growth rate of about 0.2 mm/yr for *Hulus Upper b *(Table 1) would imply an age of more than a thousand years for the oldest sample (IV), but its radiocarbon age contradicts this (Fig. 4). We have assumed that there are underlying limits to long-term variation in growth rate; it is not expected to be persistently high, and the bell-shaped curve should therefore have a shallow slope with a low max, rather than a steep slope with a high max. This assumption implies that the long-term environmental factors are approximately constant and they generate the characteristic water-limited conditions of desert environments. However, short term variability in growth is expected and not in conflict with long-term trends of the growth curve model [27].

Since only a few points are used in the non-linear regression and the location of core pith was estimated rather than observed, the age scenarios are vulnerable to errors and variations in input data (midpoint vs. other estimates; 1 vs. 2 sigma ranges). Evidently, estimates of age do not claim to be accurate, but should rather be taken as indications of possible, approximate age. This approximate age will, however, represent an underestimate because we have considered neither the time taken to grow from seedling to sampling height (50 cm) nor the effect of growth suppression due to browsing and/or drought on saplings. These factors probably add decades to tree ages [26].

An obvious question is how our estimates of longevity and growth relate to other estimates from trees growing in similar conditions. Generally, long-term data on individual trees and therefore direct age estimates are lacking. Indirect methods such as those based on mortality rates or size-age relationships from periodic size increment measurements avoid the problem of long-term data access, but such estimates may be very inaccurate [28]. Nevertheless, the conventional wisdom that growth is slow and life is long in perennial desert plants seems to be supported by increasing evidence [29]. Some tree species have lifespans of more than a thousand years [30,31]. High estimates (>1000 yr) have also been reported for savanna trees [6], but newer research based on ^14^C dating estimates the age of some *A. nigrescens *and *A. nilotica *individuals to be less than 100 years [32]. Longevity estimates of greater than 100 years are cast in the form of general statements (150 y, Serengeti, [33]), based on mortality rates (>> 100 y, Mojave desert, [29]) and on repeat photography (> 100 y, Arizona, [34]). However, low age estimates of only about 40 years have also been proposed [7,8]. Gourlay [7] concluded that acacias are far younger than commonly believed; but the majority of the trees studied grew under an annual rainfall regime, under protected conditions (in the botanical garden, Harare, Zimbabwe), and perhaps under the influence of underground water seepage from nearby irrigated areas. This estimate should therefore not be generalized for acacias as such. Ward and Rohner [8], studying *A. raddiana *in the Negev, Israel, based their estimate on a size-age relationship from the Serengeti. Newer data from the Negev show that large trees are more probably close to 200 years old [35]. This estimate is based on mean incremental trunk circumference growth, measured over two consecutive 1-year periods. Rain conditions varied greatly for the two periods, and reported statistics suggest annual radial growth of 0.1 mm (dry year) and 2.6 mm (year with rainfall) for a middle sized tree (100 cm circumference). Other growth rate estimates for acacias growing in the arid tropics vary from 2 mm/yr in Southern Turkana (mean annual rainfall: 300 mm) to 14 mm/yr in Serengeti (mean annual rainfall: 500 mm; [7,26] and references therein). Compared to these estimates and taking into account the hyper-arid conditions in the ED (rainfall < 30 mm/yr), our estimates of both growth (0.2 to 2.4 mm/yr) and longevity (200 – 650 years) lie within the ranges cited above.

Narrow, parenchymatic bands like those present in all our *A. tortilis *wood cores have been successfully used as time markers for dendrochronological dating and in climatological studies elsewhere [7,16], but such methods do not seem to be applicable to *A. tortilis *wood from the ED. Bands are not formed regularly, nor do the band patterns of trees correlate with each other, either between or within sites.

Formation of bands is considered to be coincidental with cambial dormancy [15,36]. Conditions that retard or interrupt growth can be regional, caused by climatic factors (e.g. temperature, soil moisture) and/or local, caused by factors operating even on individual trees (e.g. disease, browsing and pollarding). Several authors found that a main factor inducing cambial dormancy is onset of water stress [13-15,36]. Regularity in surface and shallow soil moisture from floods or rainfall events is not expected in the ED since rainfall is highly irregular. Consequently, regular fluctuations in deeper soil moisture are not to be expected. Temperature and phenology are also related to cambial activity and band formation [7,13,37]. In the Negev, Israel, low activity or dormancy in *A. raddiana *takes place during the cold winter [37]. In the ED too there are seasonal temperature fluctuations [23], and one might therefore expect regular band formation, in particular at high altitudes with low winter temperatures such as at Hulus Upper. However, the lack of regular band formation suggests that temperature in the ED does not produce significant regular fluctuations or is not low enough to produce a period of persistently low temperature during the winter [37].

That growth conditions vary between sites is indicated by the lack of between site band correlations. However, within sites, regional factors are expected to influence all trees in a similar manner, unless they are significantly modified by local landscape heterogeneity. The cores showing highest correlation (*Sukkari a *&*b*; *Jimal c *&*b*) seem to grow under homogenous conditions since these trees lie at short distances from one another and in a level, open part of the wadi (Fig. 2). For the same reasons homogeneity also seems to be present at Muwaylhah, but there correlation is poor between cores from neighbouring trees (*a*, *b *and *c*; *d *and *e*).

As for site homogeneity caused by soil moisture conditions, the root architecture of trees is important. Globally, rooting depth increases with aridity and roots can occupy large volumes [24,38]. Neighbouring trees should therefore draw on similar resources. However, there are also indications that within arid zones a specific rainfall regime might cause one species to invest more either in side root growth if rainfall occurs regularly or in vertical tap root growth if rainfall is more variable [39]. In Israel it has been reported that acacias are more dependent upon surface water and have shallower roots than previously believed [40,41]. It should also be taken into account that local topography modifies water run-off and therefore soil moisture conditions. This raises questions about the rooting structure of individual trees at a specific site and even at a specific location within a site. It also highlights the need for more studies on the root-root and root-water interactions in arid ecosystems.

As for poor band correlation, factors making band identification difficult must be acknowledged, as summarised by Martin and Moss [26]. Taking this into account, a study from Marsabit district, Kenya, concluded that growth limiting factors were highly variable and not simply related to climate, and further that browsing and lopping were likely to be particularly significant (as referred in [26]). Trees are widely managed also in the ED. Since our results too suggest that factors other than climate and soil moisture are important for band formation, browsing and pollarding in particular should be considered.

Pollarding is performed at irregular intervals, only on mature individuals and mainly to provide fodder for domestic animals [2]; however, in some cases nomads pollard trees to "cure" individuals that show signs of withering. Browsing, on the other hand, is a continuous disturbance. Its impact on mature trees is less dramatic since animals are herded rapidly from one tree to the next, and their reach is limited to lower heights and to the margins of the canopy. For saplings, however, the effect of browsing can be severe [26]. This suggests that browsing might influence band formation in sapling stages, while pollarding could be a factor in mature stages.

Both browsing and pollarding remove green biomass. In severe cases vital processes such as assimilation and transpiration might be retarded or stop completely. When either of these processes is reduced, the water balance of the tree is probably disturbed, perhaps to such a degree that bands form. While water stress and band formation are found to be simultaneous, the causal relationship and physiological and anatomical processes have not been described, and the function of the abundant calcium oxalate crystals in bands in *Acacia *is not properly understood (but see [36,42] and references there). This should be further investigated to better understand band formation in *A. tortilis *under hyper-arid conditions. A key question is whether band formation is always related to cambial dormancy, and how long periods without growth might be. This is also of importance for describing growth, and consequently for making reliable age estimates of desert trees.

## Conclusion

The post-bomb ^14^C content of *A. tortilis *wood provide valuable information about tree growth and is required to assess the age scenario approach adopted here. Only several dated samples from one core can give more reliable age estimates for *A. tortilis*. It is evident that the approach preliminarily explored in the present study needs to be extended to larger datasets. Also alternative methods dealing with whole populations should be further investigated [28].

The possibilities for dendrochronological studies based on *A. tortilis *from the ED are poor. However, marginal parenchymatic bands can give insight into fine scale variation in growth conditions and the past management of trees. In order to exploit this properly, the mechanisms of band formation should be further studied. Current results also suggest looking further into root-root and root-water interactions. Based on band patterns, the growth of *A. tortilis *seems to vary greatly not only temporally but also spatially, suggesting that the landscape heterogeneity is much greater than often assumed for a seemingly homogenous and harsh desert environment. Management strategies should take this into account. The great longevity (200 – 650 y) and slow growth (0.2 – 2.4 mm/y) indicated by our results also show that special care should be taken for the tree population, particularly at sites where mortality is high and recruitment is poor [43].

## Methods

### Core sampling and preparation

Destructive wood section sampling was avoided out of respect for local management traditions and because the *A. tortilis *population is decreasing [43]. Within the study area we selected seven sites, reflecting variation in hydrological and topographical conditions (Fig. 1). From these sites, 17 increment cores were sampled in February and March, 2003 (Fig. 2).

*A. tortilis *has extremely hard wood, so we developed special steel corers designed to withstand the high torque. These were powered by a rechargeable electrical drill capable of taking cores up to 300 mm long and with diameters of either 20 or 13 mm. The wide diameter was chosen to allow for micro size sampling of material for ^14^C dating. All samples were taken at 50 cm height above ground wherever possible. Samples were dried in field, using plastic tubes filled with silica gel that was replaced regularly.

All cores were sanded to high clarity and investigated macro- and microscopically. Narrow marginal parenchymatic bands, also referred to as *bands *in the following, were identifiable in all samples. In a microtome section investigated crystals were identified along these bands [36,42]. The pith is missing in the majority of the cores sampled, either because the heartwood was rotten or because eccentric or asymmetric growth made it difficult to estimate pith location. When missing, we estimated pith relative to the cambium by projecting the band- and wood anatomical pattern of a core onto an ideal, centric scheme until maximum correlation between patterns was found.

Cores were scanned at high resolution (1500 dpi), creating images to be analysed in LingoVision 5.1^© ^RINNTECH. Distance between bands and exact locations of ^14^C samples were measured to the nearest 0.01 mm. Two analysts made the measurements and disputed bands were re-examined for a final, agreed decision. All measurements were made perpendicularly to the bands.

### ^14^C analyses

Wood for accelerator mass spectroscopy (AMS) dating was sampled as drilling dust (at least 40 mg) by a 1 mm drill. To eliminate younger matter such as radially transported resin, the cellulose was extracted and precipitated from the drilled material [44]. Cellulose extraction and AMS preparation was done at the Radiological Dating Laboratory in Trondheim, Norway, and AMS dating at the Ångström Laboratory in Uppsala, Sweden. Radiocarbon age is calibrated in OxCal Version 3.10 (Copyright^© ^C. Bronk Ramsey 2005). The hyperactive ^14^C- content of wood samples is reported according to [45] and are calibrated in CALIBomb [46], using the Northern Hemisphere zone 2 dataset, smoothing 1 ("lifespan" of sample in years) and default resolution.

A total of 22 wood samples were dated (Fig. 3). For each site the longest core was selected and a sample extracted from as near as possible to the pith. With the remaining 15 samples, we aimed at the 1964-peak in the post-bomb curve for estimation of recent growth rate. These samples were extracted from four of the seven cores, from *Umm Dihaysi a*, *Muwaylhah b*, *Hulus Upper b *and *Jimal a*. Sample locations within cores were selected according to preliminary indications of possible growth rates based on ^14^C measurements from a cross section taken from a test tree.

A hyperactive sample has two possible dates, i.e. on either side of the bomb peak (1964); but in a sequence of samples from each core, one of these ranges can be eliminated. Therefore, we report only possible ages of wood formation. We refer to 1 sigma ranges or their midpoints, unless otherwise mentioned. The intervening growth rate is estimated when two or more samples from one core were hyperactive. If indicated by growth rates a more appropriate smoothing factor was chosen (cf. above).

The hyperactive samples provide a time frame for individual cores. Based on this we tested band regularity. We fitted a linear regression model to the number of bands against the time intervals.

### Age scenarios

We constructed age scenarios to give possible age estimates for the trees studied. Scenarios are used as an alternative to wiggle matching, which requires several dated samples, preferably together with information about their temporal relation [18,47]. We derive age scenarios from the bomb peak dates and possible combinations of dates of older samples, based on the midpoints of calendar ranges according to 1 sigma radiocarbon ages. The cumulative pattern of tree growth is well described by a sigmoid model [27]. We apply the Gompertz model (Equation 1a), which is fitted to our data by a non-linear least squares regression. The oldest sample is by definition set to be formed in year 0, and the positions of the samples are measured from the (estimated) pith. The tree establishment date is defined as the year in which growth has accumulated to at least a radius of 1 mm (in the height of sampling). Differential functions are derived (Equation 1b), displaying growth rate at any given time.

#### Equation 1 – Gompertz equation

(a) Integral form and (b) differential form; *y *= tree radial size, *y' *= size increment, *t *= age and *a*, *b *and *c *= parameters of the following equation:

a) y=ae−be−ctb) y'=abce−cte−be−ct
 MathType@MTEF@5@5@+=feaafiart1ev1aaatCvAUfKttLearuWrP9MDH5MBPbIqV92AaeXatLxBI9gBaebbnrfifHhDYfgasaacH8akY=wiFfYdH8Gipec8Eeeu0xXdbba9frFj0=OqFfea0dXdd9vqai=hGuQ8kuc9pgc9s8qqaq=dirpe0xb9q8qiLsFr0=vr0=vr0dc8meaabaqaciaacaGaaeqabaqabeGadaaakqaabeqaaGqabiab=fgaHjab=LcaPiabbccaGiabdMha5jabg2da9iabdggaHjabdwgaLnaaCaaaleqabaGaeyOeI0IaemOyaiMaemyzau2aaWbaaWqabeaacqGHsislcqWGJbWycqWG0baDaaaaaaGcbaGae8NyaiMae8xkaKIaeeiiaaIaemyEaKNaei4jaCIaeyypa0JaemyyaeMaemOyaiMaem4yamMaemyzau2aaWbaaSqabeaacqGHsislcqWGJbWycqWG0baDaaGccqWGLbqzdaahaaWcbeqaaiabgkHiTiabdkgaIjabdwgaLnaaCaaameqabaGaeyOeI0Iaem4yamMaemiDaqhaaaaaaaaa@54A4@

We assess these scenarios based on agreement between acquired knowledge about present growth and model characteristics. Because the underlying, environmental constraints in the harsh desert environment are approximately constant in space and time, we expect that long-term growth rate is comparable to present growth rates. The Gompertz parameter *a *indicates the maximum possible trunk radius of the tree in question. We assume that a tree had not stopped growing at the time of sampling, and would continue to grow in the near future; i.e. that the asymptotic value would be greater than the present length of the core. In distinguishing likely from less likely scenarios this cannot be used as a strict criterion since the actual pith location is only estimated (cf. above).

### Growth conditions and band patterns

We expect that trees growing under similar conditions, i.e. within sites, will exhibit very similar relative patterns of bands. Thus the correlation *between *band sequences should be high, and higher than correlations *among *sites. For correlations among sites we used all the cores from neighbouring sites (n = 32).

If correlation within sites is low, and lower than for cores among sites, this might indicate either that soil moisture conditions varied within site or that other factors induced band formation, e.g. browsing and lopping [26]. We try to remove the effect of such factors by correlating short segments within the whole cores and by shifting segments successively in either direction to allow for missing or false bands. Within sites we expect that such short segments will increase the proportion of significantly correlated segments (see below). Among sites no such effect is expected.

Because sample size must exceed a lower limit to detect a significant correlation, the shortest segment length tested is 10 bands. We also test segments of 15 and 20 bands. Each segment of core *1 *(the longest core) is tested against the same segment of core *2*, defined relative to the cambium. Segments of core *2 *are shifted up to 5 bands in either direction. The best correlation (of 11 tested) is reported for each segment of core *1*. Since segments lengths are < 50, we use the z* transformation for the calculation of t-values, testing the null hypothesis that two variables are uncorrelated [48]. The proportion of significant positively correlated segments is calculated for each set of cores. We test whether these proportions are significantly different for the three segment lengths, both within and among sites. A generalized linear model with a quasibinomial error structure (to allow for overdispersion) and a logit link is fitted [49].

Because there are more dense bands than widely separated ones, measurements are log-transformed in advance of correlation analysis. Standardisation, removing long-term trends in growth, was not done because the time intervals between bands are unknown and variable [50]. All statistical analyses were done in R 2.2.1 [51].

## Authors' contributions

GLA and KK jointly carried out the fieldwork and prepared and analysed the cores. KK designed the equipment and contributed to the finalisation of the manuscript. GLA did the statistical analyses and drafted and finalised the manuscript. Both authors read and approved the final manuscript.
